# Content prevalence is not adolescent exposure in TikTok influencer food marketing surveillance

**DOI:** 10.1017/S1368980026102213

**Published:** 2026-02-26

**Authors:** Yihan Hu

**Affiliations:** MRC Epidemiology Unit, Institute of Metabolic Science, University of Cambridge, UK

Dear Editor,

We read with interest the recent *Public Health Nutrition* accepted manuscript examining the nutritional quality of food and beverage products featured in TikTok influencer content^(1)^. The study provides timely surveillance of an increasingly influential marketing environment, with a transparent coding workflow and clear reporting of product-level healthfulness. However, one interpretive distinction warrants stronger emphasis: content prevalence is not equivalent to adolescents’ exposure. On algorithmic platforms, such as TikTok, what is posted by highly visible creators does not necessarily map onto what adolescents actually see, re-encounter or engage with – a distinction that is pivotal for policy interpretation.

TikTok is fundamentally an algorithm-driven distribution system. Individual exposure is shaped by a combination of reach (views), engagement dynamics and audience composition, all mediated by personalised recommendation systems^(2)^. As a result, estimating adolescents’ exposure requires information beyond the frequency of product appearances in sampled content. Sampling a fixed number of posts from ‘top’ influencers provides a useful snapshot of high-visibility content^(1)^, but it cannot independently infer youth-specific reach without audience demographics or view-weighted exposure metrics. This limitation is further amplified by several common yet consequential coding decisions. First, coding only the ‘most prominent’ product per video may undercount marketing density when creators feature multiple items within a single post (e.g., a branded drink alongside a dessert or takeaway meal), a pattern documented in adjacent influencer-content audits^(3)^. Second, the assumption of adolescent reach based on general platform popularity risks conflating overall visibility with youth-specific exposure^(2)^. Third, nutritional profiling challenges may be non-random: unbranded or composite foods are more likely to have uncertain nutrient information, which could systematically affect branded–unbranded comparisons if missingness is differential^(1)^. Together, these factors complicate any exposure-informed interpretation of content prevalence estimates.

We suggest three low-cost, future-oriented enhancements that could strengthen inference while preserving feasibility for ongoing surveillance. First, weight prevalence estimates by views (and ideally youth views where available) to better approximate the distribution of potential exposure across posts; even simple view-weighted estimates can reveal whether a small number of highly viewed videos disproportionately drive exposure. Second, implement bounded sensitivity analyses for missing or uncertain nutrient profiling, particularly among unbranded or composite foods, to transparently show how robust branded–unbranded contrasts are to differential missingness. Third, audit multi-product appearances in a small random subset by coding the top two products (or ‘any product’) per video, enabling quantification of undercounting due to ‘one product per video’ rules^(3)^.

Finally, we agree that influencer marketing is a plausible policy concern, supported by experimental evidence that exposure to unhealthy influencer-promoted foods can increase children’s immediate food intake^(4,5)^. Nonetheless, we encourage a cautious stepwise interpretation: surveillance of content prevalence should motivate improved exposure estimation, which in turn supports evidence-based regulation. Explicitly separating (i) featured-product prevalence, (ii) adolescent exposure and (iii) behavioural impact would avoid interpretive overreach and further strengthen the policy relevance of this important work.

Sincerely, Yihan Hu
